# Thermodynamic Evaluation and Optimization of the CaO-TiO_2_-SiO_2_ Ternary System

**DOI:** 10.3390/ma18194448

**Published:** 2025-09-23

**Authors:** Lideng Ye, Chenbo Li, Ziqian Wang, Junfeng Wu, Wenqing Zhao, Ligang Zhang, Libin Liu

**Affiliations:** School of Material Science and Engineering, Central South University, Changsha 410083, China

**Keywords:** CALPHAD, CaO-TiO_2_-SiO_2_ system, thermodynamic optimization, phase relation

## Abstract

The thermodynamic equilibrium of the CaO-TiO_2_-SiO_2_ system plays a crucial role in the design of ceramic materials. CaO-TiO_2_-SiO_2_ ternary systems were thermodynamically evaluated and optimized using the CALculation of PHAse Diagram (CALPHAD) method. The liquid phase was modeled with the ionic two-sublattice model, represented as (Ca^+2^,Ti^+2^,Ti^+3^)_P_(O^−2^,Va,O,TiO_2_,SiO_2_,SiO_4_^−4^)_Q_, and the ternary compound was described using the compound energy formalism (CEF) model. A set of self-consistent thermodynamic parameters was obtained for the CaO-TiO_2_-SiO_2_ system. The complete parameters are listed, which reliably could describe the phase equilibria and thermodynamic properties of the ternary system. This thermodynamic description provides a valuable foundation for developing larger thermodynamic databases for multicomponent silicate systems.

## 1. Introduction

CaO-SiO_2_-based glasses, constituting a fundamental silicate glass system, continue to be studied extensively due to the tunable physicochemical properties, favorable biocompatibility, and relatively low cost. Significant research interest is sustained in fields such as construction, photovoltaics, biomedicine, low-carbon materials and particularly in the development of glass-ceramics and opacified glazes [1]. The addition of modifiers like B_2_O_3_ and TiO_2_ to CaO-SiO_2_-based glasses enables the production of glass-ceramics exhibiting diverse crystalline phases and superior properties [2,3,4].

A prominent application of the CaO-TiO_2_-SiO_2_ system is in the development of zirconium-free opaque glazes [5,6]. Conventional zirconium silicate opacifiers are increasingly phased out due to concerns over natural radioactivity and high cost. TiO_2_ represents an effective alternative opacifier. However, its direct introduction often leads to undesirable yellowing of the glaze, primarily caused by the formation of rutile TiO_2_. Recent studies have demonstrated that optimizing the composition and heat treatment to promote the oriented crystallization of titanite (CaTiSiO_5_) instead of rutile can effectively mitigate yellowing while achieving superior whiteness and a higher refractive index. This highlights the critical need for a thorough understanding of the phase equilibria within the CaO-TiO_2_-SiO_2_ system to rationally design these materials. Furthermore, this ternary system serves as the fundamental foundation for more complex multi-component systems (e.g., CaO-TiO_2_-SiO_2_-B_2_O_3_) used in advanced glass-ceramics. Although glasses are non-equilibrium materials, their controlled crystallization—essential for glass-ceramic production—is intrinsically guided by the underlying equilibrium phase diagram. The diagram predicts the stable crystalline phases that form upon heat treatment, making accurate thermodynamic data a prerequisite for optimizing processing conditions and final properties. Consequently, the thorough understanding of the phase equilibria in the CaO-SiO_2_-TiO_2_-B_2_O_3_ system is essential for designing novel high-performance glass-ceramics and enhancing the properties of existing materials. The knowledge provides the fundamental theoretical underpinning for industrial practices in glass-ceramic manufacturing and related fields. To efficiently acquire phase equilibrium data, thermodynamic calculations should be performed using appropriate databases and specialized software [7,8].

The CALPHAD method facilitates the development of self-consistent thermodynamic databases [9]. When integrated with appropriate computational tools, this approach significantly reduces the time and cost involved in materials development, thereby offering valuable guidance for the design of advanced materials. Establishing a reliable multicomponent thermodynamic database for the CaO-SiO_2_-TiO_2_-B_2_O_3_ quaternary system necessitates accurate thermodynamic descriptions of all constituent ternary subsystems. Therefore, the thermodynamic parameters of the CaO-TiO_2_-SiO_2_ ternary system are indispensable for constructing a self-consistent multicomponent thermodynamic database for the CaO-SiO_2_-TiO_2_-B_2_O_3_ system.

This work critically assesses previous experimental investigations and thermodynamic optimization results for the CaO-TiO_2_-SiO_2_ ternary system and its constituent binary subsystems. Based on earlier work, a comprehensive re-optimization of the CaO-TiO_2_-SiO_2_ system was conducted using the two-sublattice ionic liquid model for the liquid phase. The compound energy formalism (CEF) was applied to model and optimize the phase CaTiSiO_5_ within the CaO-TiO_2_-SiO_2_ system. This work yields a set of self-consistent thermodynamic parameters that reliably reproduce the phase equilibria and thermodynamic properties of the system.

## 2. Review of Literature Data

### 2.1. CaO-TiO_2_ System

In the 1930s, phase equilibria on the TiO_2_-rich side of the CaO-TiO_2_ system were experimentally determined by Umezu [10], Fukusima [11], and Von Wartenberg et al. [12]. The existence of three intermediate phases CaTiO_3_, Ca_3_Ti_2_O_7_, and Ca_2_TiO_4_ along with two eutectic reactions was demonstrated in their work. In 1954, the experimental phase diagram data for the CaO-TiO_2_ system were nearly simultaneously reported by DeVries et al. [13] and Coughanour et al. [14] using X-ray diffraction (XRD) analysis. The presence of the CaTiO_3_ and Ca_3_Ti_2_O_7_ phases was confirmed through these studies. Based on XRD patterns published by DeVries et al. [13], Roth [15] synthesized a sample with a molar ratio of CaO:TiO_2_ = 4:3 via solid-state reaction and identified the Ca_4_Ti_3_O_10_ phase for the first time. Roth [15] determined incongruent melting temperatures of 2013 K and 2028 K for Ca_3_Ti_2_O_7_ and Ca_4_Ti_3_O_10_, respectively. Subsequent detailed studies of liquidus relations in the CaO–TiO_2_ system by Jongejan and Wilkins [16] and Kimura and Muan [17] further confirmed the existence of Ca_4_Ti_3_O_10_. In 1976, Tulgar [18] reported an additional compound, Ca_5_Ti_4_O_13_. More recently, Gong et al. [19] prepared a series of specimens with varying CaO:TiO_2_ ratios through solid-state synthesis and experimentally verified that CaTiO_3_, Ca_3_Ti_2_O_7_, and Ca_4_Ti_3_O_10_ are thermodynamically stable in the CaO-TiO_2_ system.

In 1946, Naylor and Cook [20] employed solution calorimetry to experimentally determine the heat content and entropy of CaTiO_3_. In 1955, King [21] measured the heat capacity of Ca_3_Ti_2_O_7_ over the temperature range 51–298 K using adiabatic calorimetry and reported its entropy at 298 K. Taylor and Schmalzried [22] performed thermodynamic measurements on CaTiO_3_ and Ca_4_Ti_3_O_10_ at 823 K using electromotive force (EMF) measurements with solid-state fluoride electrolyte cells. The formation enthalpy of the CaTiO_3_ was determined by Takayama-Muromachi and Navrotsky [23] via solution calorimetry. Guyot et al. [24] investigated the heat content of CaTiO_3_ via drop calorimetry. In 1999, Woodfield et al. [25] obtained heat capacity data for CaTiO_3_ using adiabatic calorimetry. That same year, Putnam et al. [26] studied its formation enthalpy using solution calorimetry. The structural transition temperature of CaTiO_3_ was reported by Ali et al. [27] and subsequently confirmed by Yashima et al. [28] through neutron diffraction analysis. Jacob and Abraham [29] determined the standard Gibbs free energies of formation for CaTiO_3_, Ca_3_Ti_2_O_7_, and Ca_4_Ti_3_O_10_ between 900 and 1250 K using solid-state cells with single-crystal CaF_2_ electrolyte. Navi et al. [30] also reported the formation enthalpy of CaTiO_3_. More recently, Gong et al. [19] prepared specimens via solid-state reaction and reported heat capacities of Ca_3_Ti_2_O_7_ and Ca_4_Ti_3_O_10_ from 300 to 1073 K, along with their standard formation enthalpies at 298 K, using a combination of differential scanning calorimetry (DSC) and solution calorimetry.

**Figure 1:**
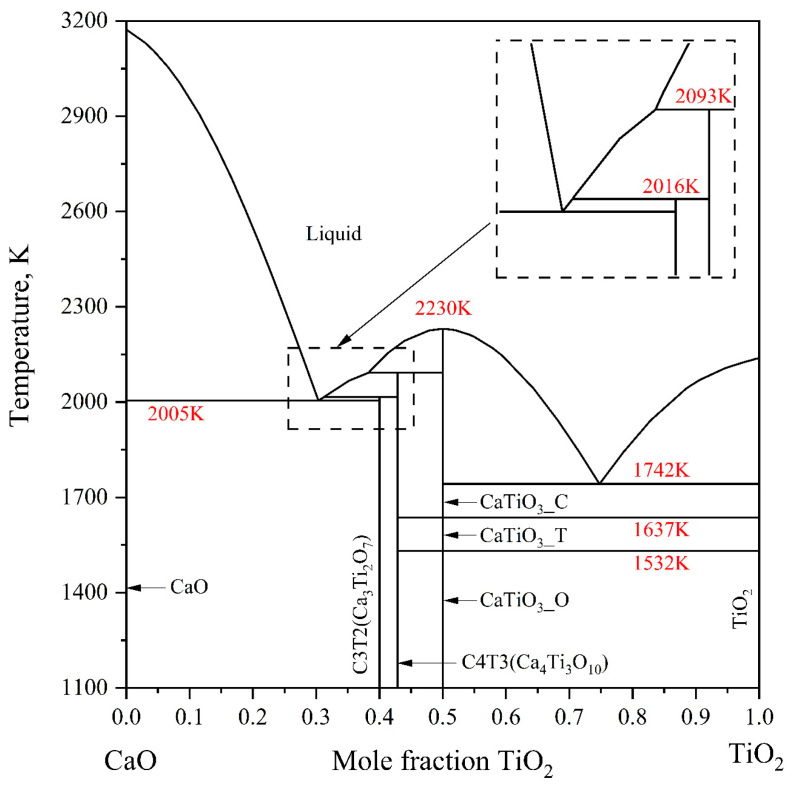
The CaO-TiO_2_ binary phase diagram evaluated by Ye et al. [34].

Kaufman [31], Kirschen et al. [32], Danek et al. [33], and Gong et al. [19] have conducted thermodynamic assessments of the CaO-TiO_2_ system and reported calculated phase diagrams. However, notable discrepancies exist among their computational results. In 1988, Kaufman [31] published a calculated phase diagram that included the compounds CaTiO_3_, Ca_3_Ti_2_O_7_, and Ca_4_Ti_3_O_10_. Nevertheless, the calculated melting point of Ca_3_Ti_2_O_7_ was higher than most experimental values, and the structural transition of CaTiO_3_ was not accounted for. Furthermore, the melting temperatures of CaO and TiO_2_ adopted by Kaufman [31] differ from those widely accepted in subsequent research. In 1999, Kirschen et al. [32] performed thermodynamic modeling of the CaO-TiO_2_ system using the Margules solution model to describe the liquid phase. The calculated phase diagram incorporated three intermediate phases: CaTiO_3_, Ca_3_Ti_2_O_7_, and Ca_5_Ti_4_O_13_. In 2002, Danek et al. [33] reported another calculated phase diagram for the same system, which included only two intermediate compounds, CaTiO_3_ and Ca_3_Ti_2_O_7_. The computed melting point of Ca_3_Ti_2_O_7_ showed a slight deviation from experimental data. Additionally, polymorphic transitions in CaTiO_3_ were omitted, and the melting temperatures used for CaO and TiO_2_ significantly diverged from the consensus values established by later researchers. Gong et al. [19] carried out a critical evaluation of experimental data from the literature and optimized the thermodynamic parameters of the CaO-TiO_2_ system using key experimental measurements. The liquid phase was modeled using the regular substitutional solution model. The calculated phase diagram and thermodynamic properties showed excellent agreement with reliable experimental data. More recently, Ye et al. [34] reoptimized the system by adopting an ionic two-sublattice model for the liquid phase. The resulting phase equilibria and thermodynamic properties are in excellent consistency with experimental findings. Therefore, the thermodynamic parameters reported by Ye et al. [34] were adopted in the present study. The calculated phase diagram for the CaO-TiO_2_ binary system is presented in Figure 1.

### 2.2. CaO-SiO_2_ System

The first complete phase diagram of the CaO-SiO_2_ binary system was established by Rankin [35] in 1915, who reported melting points of 2399 K for Ca_2_SiO_4_ and 1813 K for CaSiO_3_, along with three eutectic points in the system. The study also demonstrated that Ca_3_SiO_5_ decomposes incongruently at 2173 K into CaO and Ca_2_SiO_4_. In 1927, Greig [36] employed quenching techniques to investigate the liquid miscibility gap in the CaO-SiO_2_ system and provided detailed liquidus data on the SiO_2_-rich side. Using similar quenching methods, Osborn [37] reported that Ca_3_Si_2_O_7_ melts peritectically at 1737 K and forms a eutectic at 1733 K. The solubility of CaO in Ca_2_SiO_4_ was experimentally determined by Trömel et al. [38] using differential thermal analysis (DTA) and high-temperature X-ray diffraction. The results indicated that Ca_2_SiO_4_ is stable only within the temperature range of 1573–2073 K. In 1979, a detailed investigation of the liquid miscibility gap in the CaO–SiO_2_ system was conducted by Tewhey et al. [39]. The activities of SiO_2_ and CaO in the binary system were experimentally determined by Kay and Taylor [40] and Sharma and Richardson [41], respectively. These findings have been widely referenced in subsequent research.

**Figure 2:**
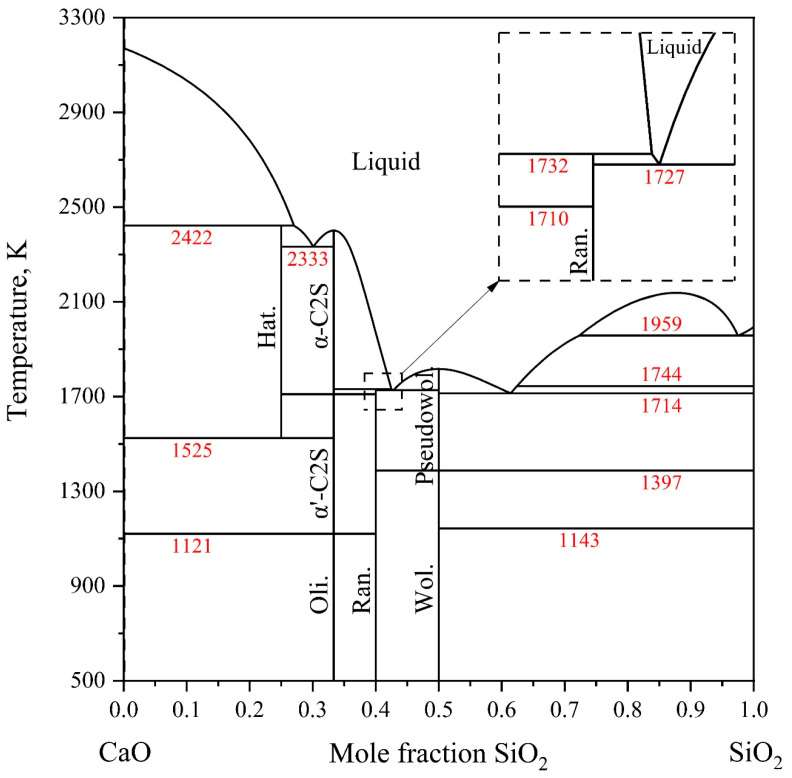
The calculated CaO-SiO_2_ binary phase diagram according to Ref. [47].

In 1990, Taylor and Dinsdale [42] conducted a thermodynamic optimization of the CaO-SiO_2_ system through a critical evaluation of available thermodynamic and phase diagram data. They employed an extended Kapoor–Frohberg cellular model to describe the liquid phase. Subsequently, Hillert et al. [43] developed a thermodynamic description using an ionic two-sublattice model for the liquid phase; however, a discrepancy was observed between their calculated liquid miscibility gap and experimental results. To improve the reliability of the thermodynamic parameters, Hillert et al. [44] reassessed the thermodynamic properties of the Ca_3_Si_2_O_7_ phase the following year. In 1994, Eriksson et al. [45] adopted the quasi-chemical model to represent the liquid phase. Shu et al. [46] utilized the Royal Institute of Technology (KTH) model for the liquid phase, though the calculated activity of SiO_2_ deviated from experimental measurements. In 1995, Huang et al. [47] performed a thermodynamic assessment using an ionic two-sublattice model, (Ca^2+^)_p_(O^2−^,SiO_4_^4−^,SiO_2_)_q_, for the liquid phase. The calculations accurately reproduced experimental phase diagrams and thermodynamic properties. This model is compatible with the thermodynamic approach adopted in the present study. Therefore, the parameters reported by Huang et al. [47] were employed in this work. The calculated binary phase diagram from their assessment is presented in Figure 2.

### 2.3. SiO_2_-TiO_2_ System

In 1933, Bunting [48] experimentally investigated phase equilibria in the SiO_2_-TiO_2_ system and reported a eutectic reaction at 8.0 at.% TiO_2_ and 1813 K. Subsequent studies by Rickers and Hummel [49] and Agamawi and White [50] also determined the eutectic reaction on the SiO_2_-rich side, though their results showed discrepancies with the data of Bunting [48]. In 1954, DeVries et al. [51] confirmed a eutectic reaction at 8.1 at.% TiO_2_ and 1823 K. Their work also established the presence of a liquid miscibility gap extending from 15 to 90.8 at.% TiO_2_ at 2053 K. In 1957, Mctaggart and Andrews [52] experimentally investigated liquid–liquid immiscibility in the system and demonstrated the coexistence of two liquid phases above 2038 K. Phase equilibria at temperatures above 2533 K were later reported by Kirschen et al. [32] and Kirillova et al. [53]. More recently, Ilatovskaia and Fabrichnaya [54] examined phase equilibria through high-temperature furnace reactions, using scanning electron microscopy (SEM) and DTA to construct the phase diagram.

**Figure 3:**
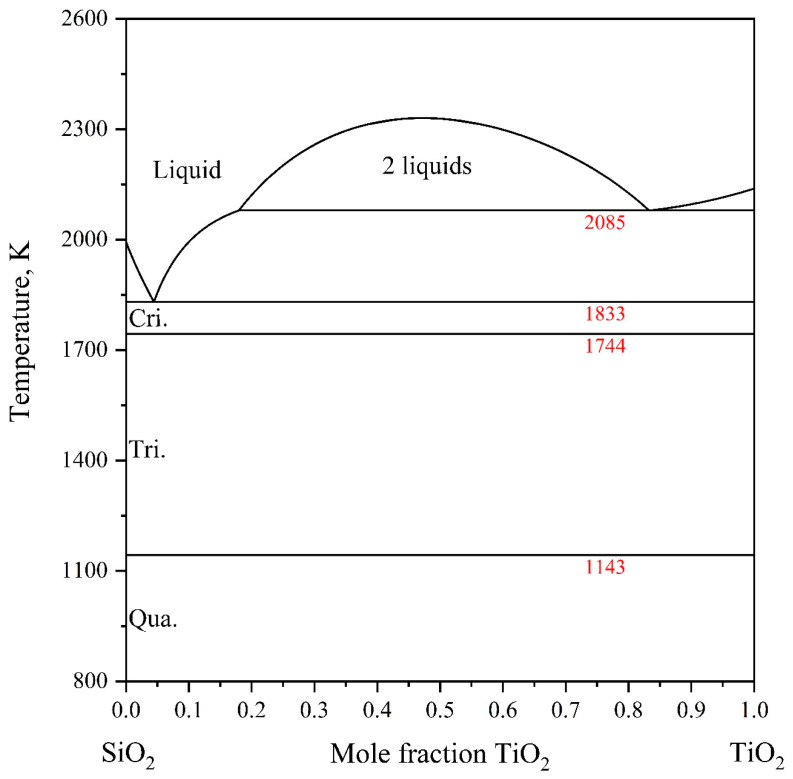
The SiO_2_-TiO_2_ binary phase diagram calculated by Ilatovskaia et al. [54].

In 1988, Kaufman [31] conducted the first thermodynamic assessment of the SiO_2_-TiO_2_ system based on experimental data for the liquid miscibility gap. Subsequently, DeCapitani and Kirschen [55] and Kirschen et al. [32] performed thermodynamic assessments using the Margules solution model for the liquid phase, assuming both temperature-independent and temperature-dependent interaction parameters. Kirillova et al. [53] reported a calculated phase diagram employing a subregular solution model for the liquid phase, grounded in key experimental measurements. In 2014, Boulay et al. [56] carried out a thermodynamic assessment that incorporated recent experimental data and adopted an ionic two-sublattice model, (Ti^+4^)_p_(O^−2^,SiO_4_^−4^,SiO_2_)_q_, for the liquid phase. However, the formulation of their liquid model deviates from currently accepted conventions. In 2022, Ilatovskaia et al. [54] reoptimized the SiO_2_-TiO_2_ system using an ionic two-sublattice model formulated as (Ti^+2^,Ti^+3^)_P_(O^−2^,SiO_4_^−4^,TiO_2_,SiO_2_)_Q_ for the liquid phase, combined with critical experimental measurements. The optimized results show excellent agreement with experimental phase diagrams. Therefore, the thermodynamic parameters reported by Ilatovskaia et al. [54] were adopted in the present study. The calculated SiO_2_-TiO_2_ binary phase diagram is presented in Figure 3.

### 2.4. CaO-TiO_2_-SiO_2_ System

In 1955, DeVries et al. [57] carried out the first systematic investigation of the phase diagram for the CaO-TiO_2_-SiO_2_ system in air. The study employed the equilibration–quenching technique along with characterization methods such as petrographic microscopy and XRD. Based on the experimental results, they constructed the liquidus projection and revealed a broad liquid immiscibility region within the system. In 1976, Panek et al. [58] experimentally determined the phase diagram of the CaTiO_3_-Ca_2_SiO_4_ pseudobinary system, particularly in the vicinity of the eutectic point, using quenching techniques and XRD. In 1998, Kirschen et al. [32,55,59] updated the primary phase fields and the immiscibility region of the CaO-TiO_2_-SiO_2_ system based on equilibration experiments conducted at 1873 K, followed by Köhler extrapolation. A significant discrepancy between their results and those of DeVries et al. [57] was identified in the stability field of CaSiO_3_. Recently, Wan et al. [60] experimentally determined the phase relations within the CaO-TiO_2_-SiO_2_ system at 1673 K under an oxygen partial pressure (P_O_2__) of 10^−10^ atm. The approach combined equilibration–quenching experiments with composition analysis of equilibrium phases using SEM coupled with energy-dispersive X-ray spectroscopy (EDS), leading to the establishment of the 1673 K isothermal section. In further work, Wan et al. [61] investigated the influence of MnO on liquid–perovskite (CaTiO_3_) phase equilibria in the same system at 1673 K. Using high-temperature equilibration–quenching techniques along with XRD, X-ray photoelectron spectroscopy (XPS), and SEM analysis, they experimentally determined the liquidus surface at that temperature. Since the present study focuses exclusively on the thermodynamic assessment of the CaO-TiO_2_-SiO_2_ system at standard atmospheric pressure, the experimental phase diagram data reported by Devries et al. [57] and Wan et al. [61] were primarily adopted during the optimization process.

In 1954, King et al. [62] reported the first measurements of the heat capacity, entropy, and heat content of sphene(CaTiSiO_5_) using adiabatic calorimetry. Their results indicated that CaTiSiO_5_ melts at 1670 K, with a molar enthalpy of fusion of 123.80 kJ·mol^−1^ and a molar entropy of fusion of 75.14 J·mol^−1^·K^−1^. Todd and Kelley [63] determined the enthalpy of formation of CaTiSiO_5_ using hydrofluoric acid solution calorimetry. Zhang et al. [64] measured the heat capacity of CaTiSiO_5_ between 300 and 900 K via differential scanning calorimetry (DSC), revealing two phase transitions near 500 K and 900 K. Xirouchakis et al. [65] re-determined the enthalpy of formation of sphene at 298 K using a 2PbO·B_2_O_3_ solvent at 702 °C. In 1999, Thieblot et al. [66] investigated the heat capacity of CaTiSiO_5_ using drop calorimetry and reported a melting temperature of 1658 ± 3 K, which is slightly lower than the value given by King et al. [62]. This result is consistent with the melting temperature range of 1648–1656 K measured by Crowe et al. [67] in 1986 using differential thermal analysis (DTA). Tangeman and Xirouchakis [68] employed DSC to determine the heat capacity of sphene from 328 to 938 K, observing a structural transition near 483 K with an associated enthalpy change of 0.196 ± 0.007 kJ·mol^−1^. Several studies have reported phase transition temperatures for CaTiSiO_5_. For example, Taylor and Brown [69] experimentally determined a transition temperature of 493 ± 20 K, corresponding to a structural change from the monoclinic space group *P*2_1_/*a* to *A*2/*a*. In 1996, Meyer et al. [70] studied the thermal behavior of CaTiSiO_5_ using infrared and Raman spectroscopy along with XRD, identifying two structural transitions at 500 K and 825 K. Additional phase transition temperatures were reported by Chrosch et al. [71] and Bismayer et al. [72]. In the present thermodynamic optimization, only two polymorphs of CaTiSiO_5_ (α and β) were considered due to the lack of thermodynamic data—such as the transition enthalpy from β to γ—for higher-temperature phases. Should a γ-CaTiSiO_5_ phase exist, its thermodynamic properties are expected to be very similar to those of β-CaTiSiO_5_.

In 1994, Pelton and Wu [73] performed a thermodynamic optimization of the CaO-TiO_2_-SiO_2_ system using the Modified Quasichemical Model to describe the liquid phase. Subsequently, Decapitani and Kirschen [32,55,59] applied the Köhler extrapolation formalism [74] to represent the miscibility gap in the system, using this method to model the Gibbs free energy of the liquid phase. Danek and Nerad [33] also conducted a thermodynamic assessment of the CaO-TiO_2_-SiO_2_ system employing the Le Chatelier-Schröder equation. However, the existing thermodynamic descriptions of the CaO-TiO_2_-SiO_2_ ternary system lack a systematic evaluation of all available experimental data, and the calculated results show significant deviations from experimental observations. Therefore, a critical reassessment of the experimental data and a comprehensive re-optimization of the thermodynamic parameters for this ternary system are necessary. In the present work, a thorough thermodynamic optimization of the CaO-TiO_2_-SiO_2_ system was carried out based on a critical review of experimental phase equilibria and thermodynamic property data, using the ionic two-sublattice model to describe the liquid phase.

## 3. Thermodynamic Modeling

Thermodynamic models of all solid phases in the CaO-TiO_2_-SiO_2_ system are listed in Table 1.

### 3.1. Unary Components

The Gibbs energies of CaO, TiO_2_ and SiO_2_ are expressed as in the following:

(1) G(T)0−HSER=a+bT+cTlnT+dT2+eT−1+fT3+gT7+hT−9

where *H^SER^* denotes the standard molar enthalpy of the pure elements (Ca, Ti, Si, and O) in the Standard Element Reference state at 298.15 K and 101,325 Pa, J·mol^−1^; *T* represents the absolute temperature in Kelvin (K); *a*~*h* are those in the model that require optimization based on experimental data. The thermodynamic parameters for the pure components CaO, TiO_2_, and SiO_2_, as utilized by Huang et al. [47] and Ilatovskaia et al. [54] were adopted in the present study.

### 3.2. Liquid Phase

The liquid phase of the CaO-TiO_2_-SiO_2_ system is described by the ionic two-sublattice liquid model with the formula (Ca^+2^,Ti^+2^,Ti^+3^)_P_(O^−2^,Va,O,TiO_2_,SiO_2_,SiO_4_^−4^)_Q_, P and Q are the number of sites on the cation and anion sublattice, respectively, that vary depending on the composition to keep electroneutrality. The Gibbs energy is represented by the expression in the following:

(2) Gmliquid−HSER= +yTi+2yO−2GTi+2:O−2liquid+yTi+3yO−2GTi+3:O−2liquid+yCa+2yO−2GCa+2:O−2liquid+yCa+2ySiO4−4GCa+2:SiO4−4liquid+QyTi+2yVaGTi+2:Valiquid+yTi+3yVaGTi+3:Valiquid+yCa+2yVaGCa+2:Valiquid+ySiO2GSiO2liquid+yOGOliquid+yTiO2GTiO2liquid+PRTyTi+2lnyTi+2+yTi+3lnyTi+3+yCa+2lnyCa+2+QRTyO−2lnyO−2+yValnyVa+ySiO2lnySiO2+ySiO4−4lnySiO4−4+yOlnyO+yTiO2lnyTiO2+GmLiquidE

where *y* represents the site fraction of each species in their own sublattices in the liquid phase; $G^{liquid}$ denotes the Gibbs energy of the endmember; and *R* is the gas constant (*R* = 8.314 J·(mol·K)^−1^). GmLiquidE is the excess Gibbs energy, which is defined as follows:

(3) GmLiquidE=+yTi+2yTi+3yO−2LTi+2,Ti+3:O−2liquid+yTi+2yO−2yVaLTi+2:O−2,Valiquid+yTi+3yO−2yTiO2LTi+3:O−2,TiO2liquid  +ySiO2yOLSiO2,Oliquid+yCa+2yO−2yOLCa+2:O−2,Oliquid+yCa+2yO−2ySiO2LCa+2:O−2,SiO2liquid  +yCa+2ySiO4−4ySiO2LCa+2:ySiO4−4,SiO2liquid+yCa+2yO−2yTiO2LCa+2:O−2,TiO2liquid  +yCa+2yO−2yTiO2ySiO2LCa+2:O−2,TiO2,SiO2liquid+yCa+2ySiO4−4yTiO2LCa+2:SiO4−4,TiO2liquid  +yCa+2ySiO4−4ySiO2yTiO2LCa+2:SiO4−4,SiO2,TiO2liquid

where *L* are the interaction parameters which utilize the Redlich–Kister polynomials in the liquid phase. The first eight interaction parameters are sourced from the works of Ilatovskaia et al. [54], Huang et al. [47], Hampl et al. [75] and Ye et al. [34] The last three parameters are assessed in the present work.

### 3.3. Binary Intermediate Compounds

Three stoichiometric intermediate compounds: Ca_3_Ti_2_O_7_, Ca_4_Ti_3_O_10_ and CaTiO_3_ were reported to exist in the CaO-TiO_2_ system. Ca_3_Ti_2_O_7_ and Ca_4_Ti_3_O_10_ are considered as stoichiometric compounds in the present work. The Gibbs energies for Ca_3_Ti_2_O_7_ and Ca_4_Ti_3_O_10_ can be described as

(4) GmCiTj−HSER=i·GCaOsolid0+j·GTiO2solid0+a+bT

where *i* and *j* represent the ratios for CaO and TiO_2_ in each formula. GCaOsolid0(=GCAOS) and GTiO2solid0(=GTIO2S) are the Gibbs energies of pure solid CaO and TiO_2_, respectively. *a* and *b* are derived from the work of Ye et al. [34], which are associated with the formation enthalpies and entropies of Ca_3_Ti_2_O_7_ and Ca_4_Ti_3_O_10_. The thermodynamic description of structural transformation of CaTiO_3_ by Gong et al. [19] is reasonable and therefore adopted in this work. There are no intermediate compounds in the TiO_2_-SiO_2_ system. The Gibbs energy expression for all solid phases in the CaO-SiO_2_ system is described by

(5) Gm(T)0−HSER=a+bT+cTlnT+dT2+eT−1

where *a*~*e* are the optimized parameters. The thermodynamic parameters for the CaO-SiO_2_ system were adopted from those reported by Huang et al. [47].

### 3.4. Ternary Intermediate Compound

In the CaO-TiO_2_-SiO_2_ system, CaTiSiO_5_ is the only ternary intermediate phase. Based on the experimental findings reported by Tangeman et al. [68], CaTiSiO_5_ undergoes a structural transition from the monoclinic space group *P*2_1_/*a* to *A*2/*a* near 500 K. In the present work, the CaTiSiO_5_ is described as a stoichiometric intermediate compound, incorporating its structural transition. Its Gibbs energy expression is given by

(6) $$G_{m}^{CTA}-H^{SER}=a+bT+cTlnT+dT^{2}+eT^{-1}+fT^{3}$$

where *a*~*f* are parameters to be optimized.

## 4. Results and Discussion

In the present work, the CaO-TiO_2_-SiO_2_ system was thermodynamically optimized based on a critical assessment of experimental data from the literature. The optimization was conducted using the PARROT module of the Thermo-Calc software (https://thermocalc.com/), which applies a least-squares method to minimize the discrepancies between calculated and experimental values. Through careful evaluation of the available experimental data, appropriate weights were assigned to each dataset to ensure a reliable optimization.

Robust thermodynamic descriptions of the constituent binary subsystems are essential for the thermodynamic modeling of ternary systems. The optimization process began by incorporating the previously established thermodynamic descriptions for the CaO-TiO_2_, CaO-SiO_2_, and TiO_2_-SiO_2_ systems reported by Ye et al. [34], Huang et al. [47], and Ilatovskaia et al. [54], respectively. Subsequently, thermodynamic parameters for relevant ternary intermediate compounds and ternary interaction parameters were introduced based on the available experimental data. The thermodynamic parameters for the ternary compound CaTiSiO_5_ were optimized based on experimental thermodynamic properties, including entropy, enthalpy of formation, heat capacity, and transition enthalpy. The ternary interaction parameters for the liquid phase were refined using experimental liquidus data. Finally, a simultaneous optimization of all parameters was carried out, resulting in a self-consistent set of thermodynamic parameters that accurately represent the CaO-TiO_2_-SiO_2_ system. The optimized thermodynamic parameters obtained in this study are summarized in Table 2.

**Figure 4:**
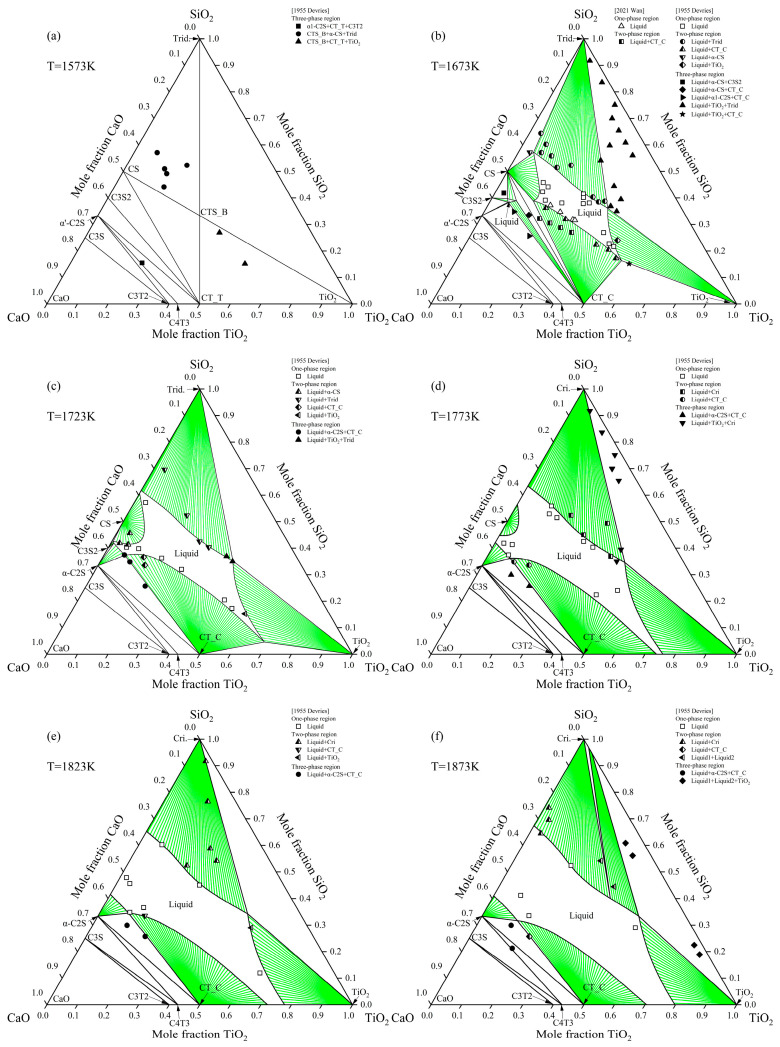
Calculated isothermal sections at (**a**) 1573, (**b**) 1673, (**c**) 1723 K, (**d**) 1773, (**e**) 1823, and (**f**) 1873 K, respectively. The experimental data are obtained from Devries et al. [57] and Wan et al. [61]. The green area represents the two-phase region.

Figure 4 presents the isothermal sections of the CaO-TiO_2_-SiO_2_ system at 1573 K, 1673 K, 1723 K, 1773 K, 1823 K, and 1873 K, along with comparisons to available experimental data reported in the literature [57,61]. The phase relations observed in all six sections are consistent with the experimental results reported by Devries et al. [57,61]. A primary difference between the present assessment and the study by Devries et al. [57] concerns the Ca_4_Ti_3_O_10_ phase, which was not detected in their work. However, subsequent experimental studies [15,19] have conclusively identified this compound. Consequently, based on a comprehensive evaluation of all available experimental data, Ca_4_Ti_3_O_10_ is accepted as a stable phase in the present thermodynamic optimization of the CaO-TiO_2_ system. It is noteworthy that while the phase assemblage interpretation differs, the high-temperature liquidus data reported by Devries et al. [57] are of high quality and are consistent with other literature sources [61]. Therefore, the present thermodynamic optimization was constrained to reproduce these reliable liquidus data, and as shown in Figure 4, the calculated liquidus exhibit excellent agreement with the experiments from [59,63].

Table 3 presents a comparison between the calculated enthalpy of formation, entropy of formation, and enthalpy of transition for CaTiSiO_5_ and available experimental data. In the CaO-TiO_2_-SiO_2_ system, the ternary intermediate phase CaTiSiO_5_ is the only one identified. It can be seen from the table that the calculated enthalpy and entropy of formation of CaTiSiO_5_ agree well with the experimental values. The structural transition in CaTiSiO_5_ was considered based on the experimental results reported by Tangeman et al. [68]. The calculated transition enthalpy of 199 J·mol^−1^ and transition temperature of 486 K show good agreement with the experimental data (transition enthalpy: 196 ± 7 J·mol^−1^; transition temperature: 483 ± 7 K) reported by Tangeman et al. [68]. The melting point of CaTiSiO_5_ obtained in this work is 1669 K. Compared to the experimental melting points (1670 K [62], 1658 ± 3 K [66], and 1648–1656 K [67]) reported in the literature for the CaTiSiO_5_ phase, the calculated value is acceptable considering the associated experimental uncertainties.

**Figure 5:**
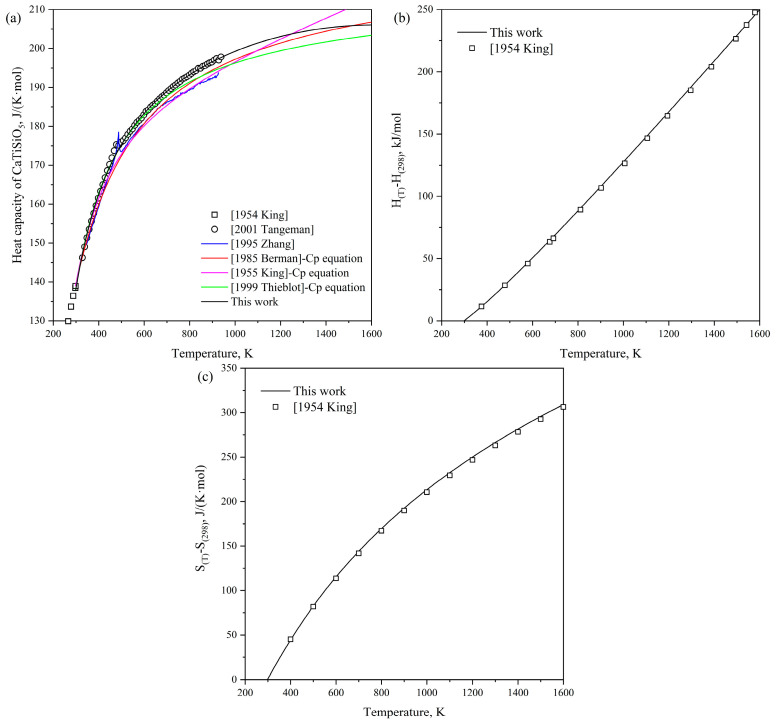
Calculated thermodynamic properties of CaTiSiO_5_ compared with the experimental data [62,68] and previous works [21,62,64,66,76]: (**a**) heat capacity; (**b**) heat content; (**c**) S_(T)_-S_(298)_.

Figure 5 displays the calculated heat capacity, heat content, and S_(T)_-S_(298)_ of the ternary phase CaTiSiO_5_, respectively, alongside experimental data from the literature [62,68]. The results calculated are found to be in good agreement with the measurements reported by King et al. [62] and Tangeman et al. [68]. Furthermore, the calculated heat capacity in this work yields a better description of the experimental data compared to previous works [62,64,66,76].

## 5. Conclusions

In this work, appropriate interaction parameters were introduced to comprehensively optimize the liquid phase and ternary compound of the system based on the existing experimental data by means of the CALPHAD approach. A set of self-consistent thermodynamic parameters was obtained, satisfactorily reproducing the experimental data of the CaO-TiO_2_-SiO_2_ ternary system. The obtained thermodynamic description is of great significance for the design of high-performance ceramic materials.

## Figures and Tables

**Table 1:** Thermodynamic models of all stable phases in the CaO-TiO_2_-SiO_2_ system.

Phase Name	Model	Reference
Liquid	(Ca^+2^,Ti^+2^,Ti^+3^)_P_(O^−2^,Va,O,TiO_2_,SiO_2_,SiO_4_^−4^)_Q_	This work
Periclase (CaO)	(Ca^+2^)_1_(O^−2^)_1_	[34]
Rutile (TiO_2_)	(Ti^+4^)_1_(O^−2^)_2_	[34]
Quartz (Qua.)	(SiO_2_)_1_	[47]
Tridymite (Tri.)	(SiO_2_)_1_	[47]
Cristobalite (Cri.)	(SiO_2_)_1_	[47]
Olivine (Oli.)	(Ca^+2^)_2_(Si^+4^)_1_(O^−2^)_4_	[47]
α′-Ca_2_SiO_4_ (α′-C2S)	(Ca^+2^)_2_(Si^+4^)_1_(O^−2^)_4_	[47]
α-Ca_2_SiO_4_ (α-C2S)	(Ca^+2^)_2_(Si^+4^)_1_(O^−2^)_4_	[47]
Larnite (Lar.)	(Ca^+2^)_2_(Si^+4^)_1_(O^−2^)_4_	[47]
Pseudowollastonite (Pseudowol.)	(Ca^+2^)_1_(Si^+4^)_1_(O^−2^)_3_	[47]
Wollastonite (Wol.)	(Ca^+2^)_1_(Si^+4^)_1_(O^−2^)_3_	[47]
Hatruite (Hat.)	(Ca^+2^)_3_(Si^+4^)_1_(O^−2^)_5_	[47]
Rankinite (Ran.)	(Ca^+2^)_3_(Si^+4^)_2_(O^−2^)_7_	[47]
CaTiO_3__O (CT_O)	(Ca^+2^)_1_(Ti^+4^)_1_(O^−2^)_3_	[34]
CaTiO_3__T (CT_T)	(Ca^+2^)_1_(Ti^+4^)_1_(O^−2^)_3_	[34]
CaTiO_3__C (CT_C)	(Ca^+2^)_1_(Ti^+4^)_1_(O^−2^)_3_	[34]
Ca_3_Ti_2_O_7_ (C3T2)	(Ca^+2^)_3_(Ti^+4^)_2_(O^−2^)_7_	[34]
Ca_4_Ti_3_O_10_ (C4T3)	(Ca^+2^)_4_(Ti^+4^)_3_(O^−2^)_10_	[34]
CaTiSiO_5_ (α-Sph.)	(Ca^+2^)_1_(Ti^+4^)_1_(Si^+4^)_1_(O^−2^)_5_	This work
CaTiSiO_5_ (β-Sph.)	(Ca^+2^)_1_(Ti^+4^)_1_(Si^+4^)_1_(O^−2^)_5_	This work

**Table 2:** Optimized thermodynamic models and parameters of CaO-TiO_2_-SiO_2_ system in the present work.

Phase	Model	Thermodynamics Parameter
Liquid	(Ca^+3^,Ti^+2^,Ti^+3^)_P_(O^−2^,Va, O,TiO_2_,SiO_2_,SiO_4_^−4^)_Q_	LCa+2:O−2,TiO2,SiO2liquid0=−993,353.075+124.275T
		LCa+2:O−2,TiO2,SiO2liquid1=−971,144+111T
		LCa+2:O−2,TiO2,SiO2liquid3=−566,824.54+128.98T
		LCa+2:SiO4−4,TiO2liquid0=+96,023−121T
		LCa+2:SiO4−4,TiO2liquid1=−263,049.615+151.255T
		LCa+2:SiO4−4,TiO2liquid3=−161,295.42+102.54T
		LCa+2:SiO4−4,SiO2,TiO2liquid0=+147,293.725−169.325T
		LCa+2:SiO4−4,SiO2,TiO2liquid1=+38,906.74−146.38T
		LCa+2:SiO4−4,SiO2,TiO2liquid3=−163,706−97T
α-Sph.	(Ca^+2^)_1_(Ti^+4^)_1_(Si^+4^)_1_(O^−2^)_5_	GCa+2:Ti+4:Si+4:O−2α−Sph.0=+GALPHASPHENE
β-Sph.	(Ca^+2^)_1_(Ti^+4^)_1_(Si^+4^)_1_(O^−2^)_5_	GCa+2:Ti+4:Si+4:O−2β−Sph.0=+GBETASPHENE
FUNCTIONS		
$\begin{array}{cc} GALPHASPHEN & =-2,666,471.78552+1078.887994T-174.50986Tln(T)-0.021445T^{2}+2.30645\times 10^{-6}T^{3}\\ & \;+2,153,777.409T^{-1}(298.15<T<6000) \end{array}$		
GBETASPHENE=+GALPHASPHENE+199−0.409465T(298.15<T<6000)		

Note: All parameter values are given in SI units.

**Table 3:** Calculated enthalpy of formation, entropy of formation, and enthalpy of transition for the CaTiSiO_5_ phases in the CaO-TiO_2_-SiO_2_ systems and their comparison with experimental data.

Properties	T, K	Calculated Value (This Work)	Experimental Value	Reference
${∆}_{f,el}H$, kJ·mol^−1^	298.15	−2598.21	−2601.4 ± 2.38	[62]
	298.15		−2602.84 ± 2.07	[63]
	298.15		−2610.13 ± 2.9	[65]
${∆}_{f,ox}H$, kJ·mol^−1^	298.15	−107.17	−110.86 ± 1.52	[62]
	298.15		−112.34 ± 1.05	[63]
	298.15		−119.59 ± 2.24	[65]
$S_{f,el}$, J·mol^−1^·K^−1^	298.15	126.31	129.2856 ± 0.8368	[62]
${∆}_{f,tr}H$, kJ·mol^−1^	-	0.199 (486 K)	0.196 ± 0.007 (483 ± 5 K)	[68]

## Data Availability

The original contributions presented in the study are included in the article; further inquiries can be directed to the corresponding authors.

## References

[1] Tulyaganov D.U., Dimitriadis K., Agathopoulos S., Fernandes H.R. (2023). Glasses and glass-ceramics in the CaO–MgO–SiO_2_ system: Diopside containing compositions—A brief review. J. Non-Cryst. Solids.

[2] Jia A.Q., Zhang W.J., Cheng X.Y., Liu Z.F. (2016). Effects of B_2_O_3_ contents on crystallization behaviors and dielectric properties of CaO-B_2_O_3_-SiO_2_ glass ceramics. Key Eng. Mater..

[3] Mukherjee D.P., Das S.K. (2014). The influence of TiO_2_ content on the properties of glass ceramics: Crystallization, microstructure and hardness. Ceram. Int..

[4] Son S., Kim K. (2023). Effect of TiO_2_ content on crystallization behavior of CaO–Al_2_O_3_–SiO_2_–ZnO glass-ceramic glaze. Ceram. Int..

[5] Lim Y., Kim K., Han K.-S. (2024). Effect of SiO_2_/TiO_2_ ratio on the crystallization behavior of CaO-TiO_2_-SiO_2_ glass-ceramic system for opaque white glaze. J. Non-Cryst. Solids.

[6] Sun S., Ding H., Ao W., Liu Y., Chang L., Zhang J. (2020). Preparation of a CaCO_3_-TiO_2_ composite based opaque glaze: Insight into the mechanism of opacification and glaze yellowing inhibition. J. Eur. Ceram. Soc..

[7] Yang J., Yan L., Ye L., Xiao G., Wang K., Liu Y., Zhang L., Liu L., Du Y. (2024). Thermodynamic evaluation and optimization of the K_2_O-Al_2_O_3_-SiO_2_ system. J. Am. Ceram. Soc..

[8] Ye L., Li C., Yang J., Xiao G., Deng Z., Liu L., Zhang L., Jiang Y. (2024). Thermodynamic Assessment of the P_2_O_5_-Na_2_O and P_2_O_5_-MgO Systems. Materials.

[9] Kattner U.R. (2016). The CALPHAD method and its role in material and process development. Tecnol. Metal. Mater. Min..

[10] Umezu S. (1930). Investigation on the iron blast furnace slag containing titanium. J. Min. Metall. Inst. Jpn..

[11] Fukusima M. (1934). The systems SiO_2_-CaO-TiO_2_, CaO·SiO_2_-CaO·SiO_2_·TiO_2_, CaO·SiO_2_·TiO_2_-TiO_2_, CaO·SiO_2_-CaO·TiO_2_, and CaO·TiO_2_-TiO_2_. Kinsoku No Kenkyu Tokyo.

[12] Von Wartenberg H., Reusch H. (1937). Melting point diagrams of highly fireproof oxides. VII. systems with CaO and BeO. Z. Fuer Anorg. Chem..

[13] Devries R., Roy R., Osborn E. (1954). Phase Equilibria in the System CaO-TiO_2_. J. Phys. Chem..

[14] Coughanour L., Roth R., Deprosse V. (1954). Phase equilibrium relations in the systems Lime-Titania. J. Res. Natl. Bur. Stand..

[15] Roth R.S. (1958). Revision of the phase equilibrium diagram of the binary system calcia-titania, showing the compound Ca_4_Ti_3_O_10_. J. Res. Natl. Bur. Stand..

[16] Jongejan A., Wilkins A. (1970). A re-examination of the system CaO-TiO_2_ at liquidus temperatures. J. Less Common Met..

[17] Kimura S., Muan A. (1971). Phase relations in the system CaO-iron oxide-TiO_2_ in air. Am. Mineral. J. Earth Planet. Mater..

[18] Tulgar H. (1976). Solid state relationships in the system calcium oxide-titanium dioxide. Istanbul Tech. Univ. Bul..

[19] Gong W., Wu L., Navrotsky A. (2018). Combined experimental and computational investigation of thermodynamics and phase equilibria in the CaO-TiO_2_ system. J. Am. Ceram. Soc..

[20] Naylor B., Cook O. (1946). High-temperature heat contents of the metatitanates of calcium, iron and Magnesium. J. Am. Chem. Soc..

[21] King E. (1955). Low-Temperature Heat Capacities and Entropies at 298.16K. of Some Titanates of Aluminum, Calcium, Lithium and Zinc. J. Am. Chem. Soc..

[22] Taylor R., Schmalzried H. (1964). The free energy of formation of some titanates, silicates, and magnesium aluminate from measurements made with galvanic cells involving solid electrolytes. J. Phys. Chem..

[23] Takayama-Muromachi E., Navrotsky A. (1988). Energetics of compounds (A^2+^B^4+^O_3_) with the perovskite structure. J. Solid State Chem..

[24] Guyot F., Richet P., Courtial P., Gillet P. (1993). High-temperature heat capacity and phase transitions of CaTiO_3_ perovskite. Phys. Chem. Miner..

[25] Woodfield B.F., Shapiro J.L., Stevens R., Boerio-Goates J., Putnam R.L., Helean K.B., Navrotsky A. (1999). Molar heat capacity and thermodynamic functions for CaTiO_3_. J. Chem. Thermodyn..

[26] Putnam R.L., Navrotsky A., Woodfield B.F., Boerio-Goates J., Shapiro J.L. (1999). Thermodynamics of formation for zirconolite (CaZrTi_2_O_7_) from T = 298.15K to T = 1500K. J. Chem. Thermodyn..

[27] Ali R., Yashima M. (2005). Space group and crystal structure of the perovskite CaTiO_3_ from 296 to 1720K. J. Solid State Chem..

[28] Yashima M., Ali R. (2009). Structural phase transition and octahedral tilting in the calcium titanate perovskite CaTiO_3_. Solid State Ion..

[29] Jacob K., Abraham K. (2009). Thermodynamic properties of calcium titanates: CaTiO_3_, Ca_4_Ti_3_O_10_, and Ca_3_Ti_2_O_7_. J. Chem. Thermodyn..

[30] Navi N.U., Shneck R.Z., Shvareva T.Y., Kimmel G., Zabicky J., Mintz M.H., Navrotsky A. (2012). Thermochemistry of (Ca_x_Sr_1-x_)TiO_3_, (Ba_x_Sr_1-x_)TiO_3_, and (Ba_x_Ca_1-x_)TiO_3_ perovskite solid solutions. J. Am. Ceram. Soc..

[31] Kaufman L. (1988). Calculation of multicomponent ceramic phase diagrams. Physica B+C.

[32] Kirschen M., Decapitani C. (1999). Experimental determination and computation of the liquid miscibility gap in the system CaO-MgO-SiO_2_-TiO_2_. J. Phase Equilib..

[33] Daněk V., Nerád I. (2002). Phase Diagram and Structure of Melts of the System CaO-TiO_2_-SiO_2_. Chem. Pap..

[34] Ye L., Li C., Yang J., Xiao G., Zhang L., Jiang Y., Liu L., Masset P.J. (2024). Critical evaluation and thermodynamic assessment of the Al_2_O_3_–TiO_2_–CaO ternary system. Ceram. Int..

[35] Rankin G. (1915). The Ternary System CaO-Al_2_O_3_-SiO_2_. Am. J. Sci..

[36] Greig J. (1927). Immiscibility in silicate melts; Part I. Am. J. Sci..

[37] Osborn E.F. (1943). The compound merwinite (3CaO·MgO·2SiO_2_) and its stability relations within the system CaO-MgO-SiO_2_ (preliminary report). J. Am. Ceram. Soc..

[38] Troemel G., Fix W., Heinke R. (1969). Hochtemperaturuntersuchungen bis 1900 °C an Calciumorthosilikat und Tricalciumsilikat. Tonind.-Ztg. Ker. Rundsch..

[39] Tewhey J.D., Pc H. (1979). The two phase region in the CaO-SiO_2_ system: Experimental data and thermodynamic analysis. Phys. Chem. Glas..

[40] Kay D., Taylor J. (1960). Activities of silica in the lime+alumina+silica system. Trans. Faraday Soc..

[41] Sharma R., Richardson F. (1962). Solubility of calcium sulphide and activities in lime-silica melts. J. Iron Steel Inst..

[42] Taylor J., Dinsdale A. (1990). Thermodynamic and phase diagram data for the CaO-SiO_2_ system. Calphad.

[43] Hillert M., Sundman B., Wang X. (1990). An assessment of the CaO-SiO_2_ system. Metall. Trans. B.

[44] Hillert M., Sundman B., Wang X., Barry T. (1991). A reevaluation op the rankinite phase in the CaO-SiO_2_ system. Calphad.

[45] Eriksson G., Wu P., Blander M., Pelton A.D. (1994). Critical evaluation and optimization of the thermodynamic properties and phase diagrams of the MnO-SiO_2_ and CaO-SiO_2_ systems. Can. Metall. Q..

[46] Shu Q., Chou K.C. (2015). Thermodynamic Modeling of CaO-CaF_2_ and CaO-SiO_2_ Systems. High Temp. Mater. Process..

[47] Huang W., Hillert M., Wang X. (1995). Thermodynamic assessment of the CaO-MgO-SiO_2_ system. Metall. Mater. Trans. A.

[48] Bunting E.N. (1933). Phase equilibria in the systems TiO_2_, TiO_2_-SiO_2_ and TiO_2_-Al_2_O_3_. Bur. Stand. J. Res..

[49] Ricker R.W., Hummel F. (1951). Reactions in the System TiO_2_-SiO_2_; revision of the phase diagram. J. Am. Ceram. Soc..

[50] Agamawi Y., White J. (1952). The System Al_2_O_3_-TiO_2_-SiO_2_. Br. Ceram. Trans..

[51] Devries R., Roy R., Osborn E. (1954). The System TiO_2_-SiO_2_. Br. Ceram. Trans..

[52] Don Mctaggart G., Andrews A. (1957). Immiscibility area in the system TiO_2_-ZrO_2_-SiO_2_. J. Am. Ceram. Soc..

[53] Kirillova S., Almjashev V., Gusarov V. (2011). Phase relationships in the SiO_2_-TiO_2_ system. Russ. J. Inorg. Chem..

[54] Ilatovskaia M., Fabrichnaya O. (2022). Liquid Immiscibility and Thermodynamic Assessment of the Al_2_O_3_-TiO_2_-SiO_2_ System. J. Phase Equilib. Diffus..

[55] Decapitani C., Kirschen M. (1998). A generalized multicomponent excess function with application to immiscible liquids in the system CaO-SiO_2_-TiO_2_. Geochim. Cosmochim. Acta.

[56] Boulay E., Nakano J., Turner S., Idrissi H., Schryvers D., Godet S. (2014). Critical assessments and thermodynamic modeling of BaO-SiO_2_ and SiO_2_-TiO_2_ systems and their extensions into liquid immiscibility in the BaO-SiO_2_-TiO_2_ system. Calphad.

[57] Devries R., Roy R., Osborn E. (1955). Phase equilibria in the system CaO-TiO_2_-SiO_2_. J. Am. Ceram. Soc..

[58] PÁnek Z., KanclÍř E., Staroň J., Kozlovský M., Palčo Š. (1976). The effect of TiO_2_ on phase transformations of periclase-spinellitic systems in the presence of silicates. Ceram.-Silik..

[59] Kirschen M., De Capitani C. (1998). Immiscible silicate liquids in the CaO-SiO_2_-TiO_2_-Al_2_O_3_ system. Schweiz. Mineral. Petrogr. Mitt..

[60] Wan X., Shi J., Klemettinen L., Chen M., Taskinen P., Jokilaakso A. (2020). Equilibrium phase relations of CaO-SiO_2_-TiO_2_ system at 1400 °C and oxygen partial pressure of 10^−10^ atm. J. Alloys Compd..

[61] Wan X., Chen M., Qiu Y., Shi J., Li J., Liu C., Taskinen P., Jokilaakso A. (2021). Influence of manganese oxide on the liquid-perovskite equilibrium in the CaO-SiO_2_-TiO_2_ system at 1400 °C in air. Ceram. Int..

[62] King E., Orr R., Bonnickson K. (1954). Low temperature heat capacity, entropy at 298.16K., and high temperature heat content of sphene (CaTiSiO_5_). J. Am. Chem. Soc..

[63] Todd S., Kelley K. (1956). Heat and Free-Energy Data for Tricalcium Dititanate, Sphene, Lithium Metatitanate, and Zinc-Titanium Spinel.

[64] Zhang M., Salje E., Bismayer U., Unruh H.-G., Wruck B., Schmidt C. (1995). Phase transition(s) in titanite CaTiSiO_5_: An infrared spectroscopic, dielectric response and heat capacity study. Phys. Chem. Miner..

[65] Xirouchakis D., Fritsch S., Putnam R.L., Navrotsky A., Lindsley D.H. (1997). Thermochemistry and the enthalpy of formation of synthetic end-member (CaTiSiO_5_) titanite. Am. Mineral..

[66] Thiéblot L., Tequi C., Richet P. (1999). High-temperature heat capacity of grossular (Ca_3_Al_2_Si_3_O_12_), enstatite (MgSiO_3_), and titanite (CaTiSiO_5_). Am. Mineral..

[67] Crowe M., Greedan J., Garrett J., Burke N., Vance E., George I. (1986). Melt-grown sphene (CaTiSiO_5_) crystals. J. Mater. Sci. Lett..

[68] Tangeman J., Xirouchakis D. (2001). High-temperature heat capacity and thermodynamic properties for end-member titanite (CaTiSiO_5_). Phys. Chem. Miner..

[69] Taylor M., Brown G. (1976). High-temperature structural study of the P21/a↔A2/a phase transition in synthetic titanite, CaTiSiO_5_. Am. Mineral..

[70] Meyer H., Zhang M., Bismayer U., Salje E., Schmidt C., Kek S., Morgenroth W., Bleser T. (1996). Phase transformation of natural titanite: An infrared, Raman spectroscopic, optical birefringence and X-ray diffraction study. Phase Transit. A Multinatl. J..

[71] Chrosch J., Bismayer U., Salje E.K. (1997). Anti-phase boundaries and phase transitions in titanite: An X-ray diffraction study. Am. Mineral..

[72] Bismayer U., Zhang M., Groat L., Salje E., Meyer H.-W. (1999). The β-γ phase transition in titanite and the isosymmetric analogue in malayaite. Phase Transit..

[73] Pelton A., Wu P. (1994). Thermodynamically Optimised Phase Diagrams of Oxide Systems.

[74] Kohler F. (1960). Estimation of the thermodynamic data for a ternary system from the corresponding binary systems. Monatshefte Fuer Chem..

[75] Hampl M., Schmid-Fetzer R. (2015). Thermodynamic description of the Ti-O system. Int. J. Mater. Res..

[76] Berman R.G., Brown T.H. (1985). Heat capacity of minerals in the system Na_2_O-K_2_O-CaO-MgO-FeO-Fe_2_O_3_-Al_2_O_3_-SiO_2_-TiO_2_-H_2_O-CO_2_: Representation, estimation, and high temperature extrapolation. Contrib. Mineral. Petrol..

